# An asymptomatic pulmonary arteriovenous fistula presenting with a Campylobacter rectus brain abscess as the initial manifestation: a case report

**DOI:** 10.3389/fmed.2026.2008073

**Published:** 2026-10-27

**Authors:** Jiarong Li, Xinxin Ji, Huanming Zhang, Yu Liao, Jianwei Li

**Affiliations:** 1 Department of General Practice, Xiangtan Central Hospital (Affiliated Hospital of Hunan University), Xiangtan, China; 2 Department of General Practice, Binzhou People's Hospital, Binzhou, China; 3 Department of Radiology, Xiangtan Central Hospital (Affiliated Hospital of Hunan University), Xiangtan, China

**Keywords:** asymptomatic, brain abscess, Campylobacter rectus, case report, pulmonary arteriovenous fistula

## Abstract

**Background:**

Brain abscess is a severe infectious disease of the central nervous system associated with high rates of mortality and morbidity. Pulmonary arteriovenous fistula (PAVF) is a rare but important cause of cryptogenic brain abscesses. The pathogens associated with PAVF-related brain abscesses are typically oral anaerobes or facultative anaerobes, most commonly non-hemolytic streptococci and *Streptococcus intermedius*. To our knowledge, no case of a monomicrobial Campylobacter rectus (C. rectus) brain abscess in a patient with asymptomatic PAVF has been reported.

**Case presentation:**

Herein, we report the case of a 57-year-old man with a *C. rectus* brain abscess in association with an asymptomatic PAVF. The patient presented to our hospital with fever and headache. Magnetic resonance (MR) imaging revealed a 5 × 2 cm brain abscess in the right temporal and occipital lobes, surrounded by extensive edema. Enhanced computed tomography (CT) scanning of the lungs revealed both nodules and PAVF. Blood culture and cardiac ultrasound failed to detect any abnormalities. Piperacillin-tazobactam and meropenem were administered for anti-infection, although the headache persisted. He was subsequently transferred to neurosurgery to resect the abscess by craniotomy; postoperative pus culture confirmed the presence of *C. rectus*.

**Conclusions:**

For brain abscesses caused by oral anaerobes such as *C. rectus*, screening for right-to-left shunting diseases such as PAVF may be considered when no other source is identified.

## 1 Introduction

Brain abscess is a life-threatening focal infection of the central nervous system and is highly susceptible to misdiagnosis due to its atypical clinical manifestations. The incidence of brain abscess has increased in the elderly, with a nationwide study in Germany reporting a 122.2% increase from 2005 to 2023 (0.9 to 2.2 per 100,000), and the highest incidence in the 65–74 year age group (3.6 per 100,000) (1). It is usually caused by hematogenous dissemination, contiguous spread, and structural abnormalities such as PAVF (2). Oral bacteria, including streptococci, staphylococci, and gram-negative enterobacteria, are currently the most common pathogens causing brain abscess (3).

*C. rectus is an obligate anaerobic, gram-negative bacterium* that colonizes the oral cavity. *C. rectus is predominantly associated* with active periodontitis, but can also enter the bloodstream through occult odontogenic foci—such as dental caries, gingivitis, and periodontitis—that may remain clinically silent without overt dental symptoms (4). Thus far, rare cases of systemic disseminated infections have been reported in the literature, including brain abscess, lung abscess, and liver abscess (5–7).

Despite limitations in epidemiologic studies, the approximate incidence of PAVF has been reported to be 2–3 per 100,000 (8). Among patients with PAVF, brain abscess has been reported in 8.3% (37/445) of cases (9), and 29 of these 37 patients (78.4%) had not been diagnosed with PAVF before their abscess (9). These findings support the view that PAVF is an uncommon but increasingly recognized risk factor for paradoxical embolization and brain abscess formation. By establishing a right-to-left shunt that bypasses pulmonary capillary filtration, PAVF may provide a direct route for pathogens from the venous circulation, including microorganisms of odontogenic origin, to enter the systemic arterial circulation and potentially reach the brain (10). In addition to brain abscess, septic emboli traversing the shunt have, rarely, been associated with infection at other systemic sites; for example, synchronous liver and brain abscesses have been reported in a patient with a PAVM (15), although such non-cerebral infections remain confined to isolated case reports.

To our knowledge, this is the first case report to describe a monomicrobial *C. rectus* brain abscess in a patient with asymptomatic PAVF. Our findings suggest that, in cryptogenic brain abscess, screening for PAVF and identification of oral pathogens may be considered even when dental symptoms are absent.

## 2 Case presentation

A 57-year-old man presented to the Department of General Practice at our hospital with a four-day history of fever and headache, and was subsequently admitted to the same department. There was a history of exposure to cold before onset, and the body temperature was not recorded. Additional symptoms included chills and nausea, with headache exacerbating upon postural changes. The patient denied cough, the production of sputum, dyspnea, weight loss, abdominal pain, diarrhea, dysuria, toothache, bone pain, limb weakness or hemiparesis, paresthesia, aphasia, or alterations in his personality. The patient had no history of epistaxis, telangiectasia, or similar family history, nor any history of dental disease or dental procedures, intravenous drug abuse, congenital heart disease, diabetes, or other immunocompromising conditions. He had no history of hypertension, hyperlipidemia, or long-term medication use. The patient was a farmer with no history of smoking or alcohol consumption. He brushed his teeth twice daily and had never visited a dentist.

Prior to admission, a complete blood count (CBC) at a local hospital revealed leukocytosis, and computed tomography (CT) of the chest revealed a mass in the upper left lung. On admission, vital signs (temperature, blood pressure, heart rate and oxygen saturation) were within normal ranges. The patient was conscious and oriented, with normal comprehension, memory, and calculation abilities. Oral examination by the general practitioner revealed poor oral hygiene with visible dental plaque and calculus, but no obvious dental caries, gingival swelling, or purulent discharge. No dental pain or mobility was noted. A formal dental evaluation was not performed. Physical examination found no evidence of cyanosis or clubbing, and cardiopulmonary, abdominal, and neurological examinations were unremarkable.

Laboratory investigations on admission were as follows: white blood cell count (10.3 × 10⁹/L), neutrophil count (7.5 × 10⁹/L), platelet count (326 × 10⁹/L), interleukin-6 (IL-6; 13 pg/mL), and C-reactive protein (CRP; 3.82 mg/L). Arterial blood gas analysis, procalcitonin (PCT), B-type natriuretic peptide (BNP), troponin T (TNT), thyroid function tests, lung cancer tumor markers, serum biochemistry, coagulation profile, respiratory pathogen panel, urinalysis, stool examination, and serological tests for syphilis, human immunodeficiency virus (HIV), hepatitis B virus (HBV), and hepatitis C virus (HCV) were all unremarkable. One set of aerobic and anaerobic blood cultures was collected before antibiotic administration. Cultures were processed using an automated blood culture system (BACTEC). Blood cultures showed no growth after 5 days of incubation. There were no abnormalities detected by electrocardiogram (ECG) and transthoracic echocardiography.

The patient initially presented with fever and headache. Given the outside hospital CT findings and infection markers, the differential diagnosis included both infection and malignancy. At the time, the pulmonary lesion was considered the most likely infectious focus, although involvement of other sites could not be excluded. To provide broad coverage against common community-acquired organisms, empirical antimicrobial therapy was initiated with piperacillin-tazobactam (4.5 g every 8 h). To clarify the nature of the pulmonary lesion and identify the infectious focus, contrast-enhanced chest and abdominal CT was performed on day two post-admission, which revealed two indeterminate pulmonary nodules and a PAVF in the right middle lobe (Figures 1, 2). The patient reported no typical PAVF-related symptoms, such as dyspnea, hemoptysis, cyanosis, or digital clubbing, consistent with an asymptomatic PAVF. After 3 days of empirical antibiotic therapy, body temperature trended downward, but the headache persisted. The observed pulmonary abnormalities and fever were insufficient to explain the concurrent neurological manifestations. To exclude intracranial infection or malignancy, neuroimaging was performed. Cranial magnetic resonance imaging (MRI) with gadolinium enhancement and diffusion-weighted imaging (DWI) on day six post-admission demonstrated a 5 × 2 cm brain abscess (Figure 3).

**Figure 1:**
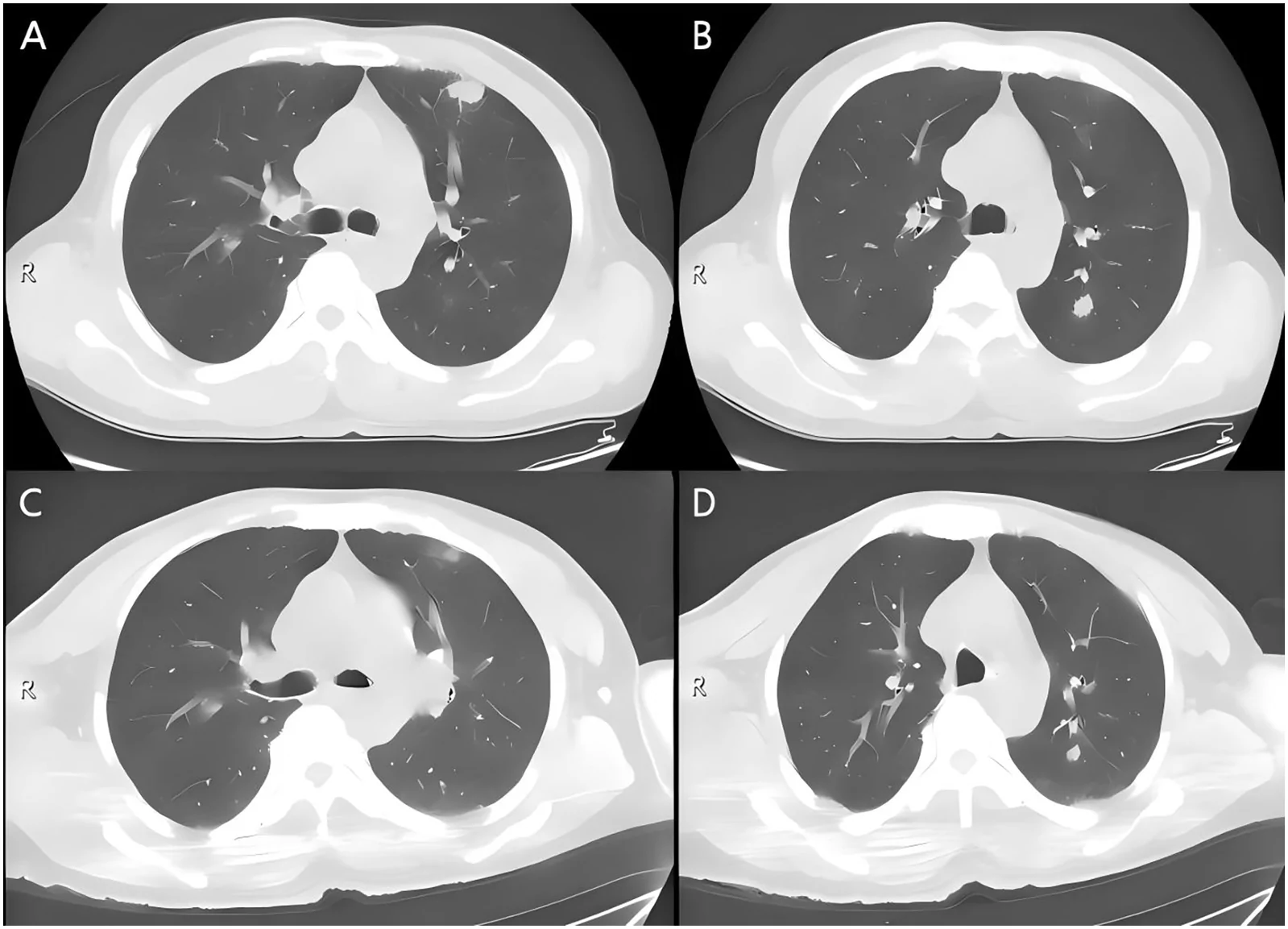
Chest CT images before **(A,B)** and after **(C,D)** treatment. **(A,B)** Pretreatment CT revealed nodular opacities in the lung. **(C,D)** Post-treatment CT demonstrated marked absorption of the lesions. CT, computed tomography.

**Figure 2:**
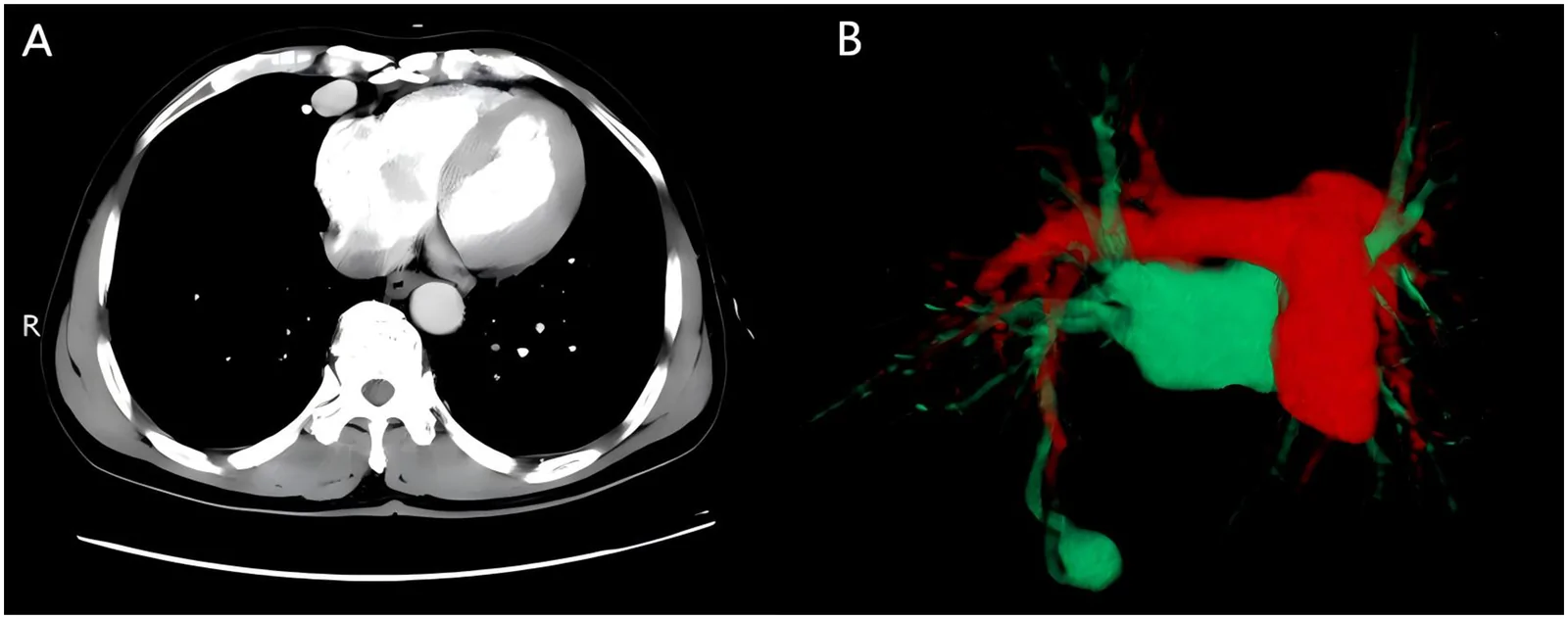
Contrast-enhanced chest CT and volume rendering (VR) reconstruction. **(A)** Contrast-enhanced chest CT revealed a pulmonary arteriovenous malformation (PAVM) in the right middle lobe. **(B)** VR reconstruction clearly demonstrated the vascular architecture of the right middle lobe PAVM, with the characteristic ‘feeding artery-aneurysmal sac-draining vein” structure.

**Figure 3:**
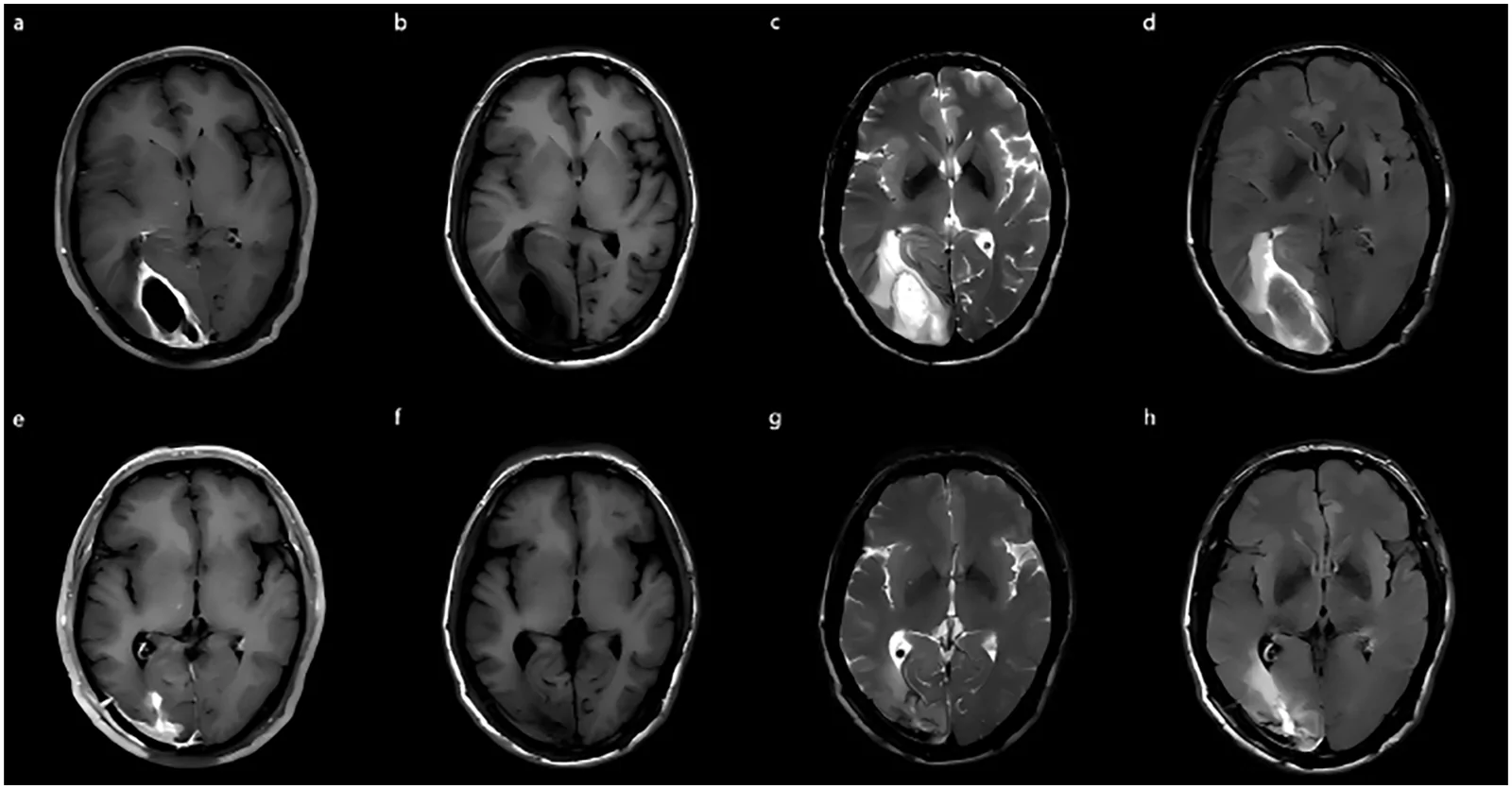
Preoperative **(A–D)** and postoperative **(E–H)** brain MRI. **(a)** Contrast-enhanced T1WI revealed thin, smooth, complete ring enhancement of the capsule. **(b)** T1WI revealed a hypointense and well-defined lesion in the right occipital lobe that was approximately 5 × 2 cm in size. **(c)** T2WI revealed a hyperintense cavity with surrounding edema. **(d)** FLAIR revealed a slightly hyperintense cavity and perilesional edema. **(e)** Contrast-enhanced T1WI at six weeks showed irregular peripheral enhancement at the surgical margin, reduced edema, and mild thickening with marked enhancement of the adjacent dura mater but with no residual abscess cavity. **(f)** T1WI revealed patchy hypointensity at the surgical site, which was decreased in extent. **(g)** T2W revealed patchy hyperintensity at the surgical bed with reduced edema. **(h)** FLAIR detected reduced hyperintensity at the surgical site. MRI, magnetic resonance imaging; T1WI, T1-weighted imaging; T2WI, T2-weighted imaging; FLAIR, fluid-attenuated inversion recovery.

Because cranial MRI showed typical findings of a brain abscess and lumbar puncture carries a risk of herniation in patients with intracranial mass lesions, cerebrospinal fluid analysis was not performed. Considering the bacterial spectrum of brain abscesses (streptococci and anaerobes) and the central nervous system penetration of the drug, the antibiotic regimen was changed to meropenem (2 g intravenously every eight hours). At the same time, the patient was transferred to the Department of Neurosurgery for further treatment. Fever resolved within three days, although the headache persisted. During this period, the departments of neurosurgery and anesthesiology assessed the surgical risk. On day nine post-admission, the patient underwent right occipital craniotomy, occipital brain abscess excision, and extended dural repair under general anesthesia. Intraoperatively, pus was collected for aerobic and anaerobic culture. The specimen was inoculated onto anaerobic blood agar plates and incubated at 37 °C under strict anaerobic conditions; incubation was extended to 4 days (routine anaerobic cultures are typically finalized at 48 h), at which time small colonies were observed. The isolate was identified as C. rectus by MALDI-TOF mass spectrometry (phenotypic identification). Regrettably, drug susceptibility testing was not feasible due to the unavailability of commercial susceptibility plates. Histopathological examination of the brain tissue revealed hemorrhage, abundant neutrophil and lymphocyte infiltration, inflammatory necrosis, and focal granulation tissue formation, consistent with suppurative inflammation.

Postoperatively, empirical therapy with meropenem (2 g intravenously every eight hours) was continued for a total of 18 days (including the postoperative period), followed by oral moxifloxacin (0.4 g once daily) for four weeks. A repeat chest CT on day twelve post-admission revealed that the inflammatory nodule had reduced in size (Figure 1). Follow-up contrast-enhanced cranial MRI at 7 weeks after admission demonstrated radiographic improvement (Figure 3). At that time, the patient was asymptomatic with no neurological symptoms, including headache. During the latest follow-up, the patient had no respiratory symptoms (dyspnea, hemoptysis, cyanosis, clubbing) or PAVF-related neurological symptoms. Transcatheter embolization of the PAVF was recommended by the Department of Interventional Radiology, but the patient declined the procedure for financial reasons. He was informed of the rationale for embolization, including the risk of recurrent brain abscess and paradoxical embolic complications if the shunt remained untreated; after considering the costs, he chose not to proceed. Figure 4 shows a timeline of the patient's diagnosis, interventions, and outcomes.

**Figure 4:**
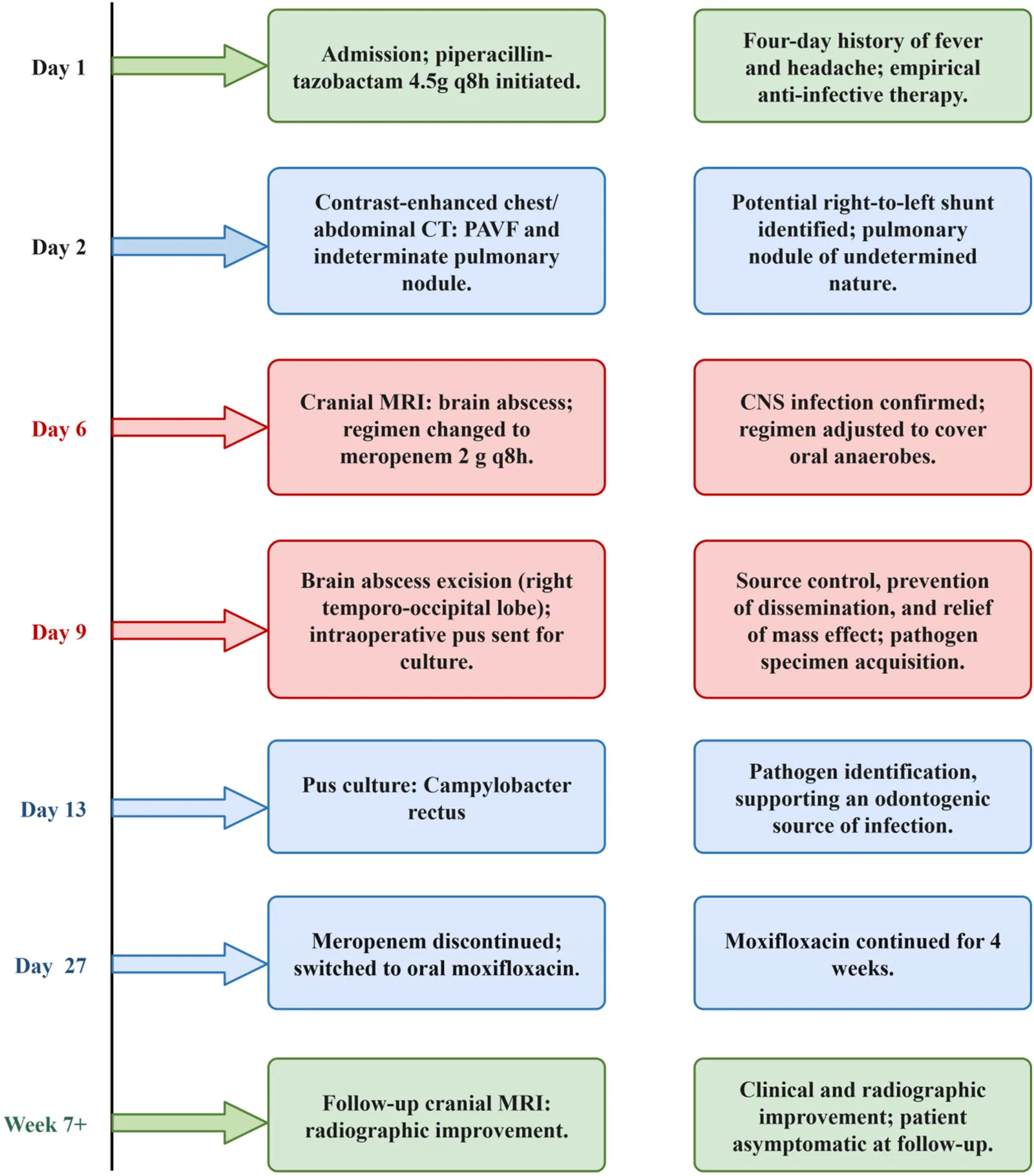
Timeline of interventions and outcomes.

## 3 Discussion

Brain abscess is usually caused by hematogenous dissemination, contiguous spread, and structural abnormalities. Comprehensive history-taking and physical examination revealed no evidence of otitis media, sinusitis, dental infection, prior cranial trauma, or neurosurgical intervention, thereby excluding contiguous spread as a plausible mechanism. In this case, blood cultures were negative, and transthoracic echocardiography showed no evidence of infective endocarditis or intracardiac shunt. Contrast-enhanced chest and abdominal CT revealed no other severe infectious foci. Therefore, common sources of hematogenous dissemination were considered unlikely. Laboratory culture of pus from the abscess led to the identification of *C. rectus*, an obligate oral commensal organism, strongly implicating the oral cavity as the most likely portal of entry. Notably, contrast-enhanced CT of the thorax revealed a PAVF. On the basis of our findings, we hypothesize a potential pathogenic pathway. Transient oral bacteremia may have seeded the pulmonary arterial circulation; subsequent shunting through the PAVF bypassed the natural microbial filtration provided by the pulmonary capillary bed, potentially permitting viable *C. rectus* to enter the systemic arterial circulation and reach the cerebral parenchyma, resulting in abscess formation.

*C. rectus* is an anaerobic and Gram-negative bacterium capable of oral colonization. A nationwide Danish cohort study of 485 adults with brain abscess established that the presence of bacteria in the oral cavity was the most frequent etiology, accounting for 59% of all cases (11); the majority of these oral-flora abscesses occurred in patients without PAVF, indicating that PAVF is not a prerequisite for such infection. However, *C. rectus* was not identified in this large cohort, thus highlighting the rarity of our case (11). Subsequent analysis, focusing specifically on the 287 patients with brain abscess related directly to the presence of bacteria in the oral cavity, found that a dental infection was identified in only 24% (68/287) of cases, and 41% (117/287) were polymicrobial (12). To date, only sporadic case reports of C. rectus brain abscesses have been published (5, 13). In a previously reported case, one patient underwent tooth extraction for a dental abscess and, despite antibiotic therapy, progressed to multiple skull base and brain abscesses 10 weeks later (5). Another case involved a 54-year-old man with diabetes mellitus who developed a *C. rectus* brain abscess secondary to periodontitis-associated pathogens (13). Both cases were characterized by documented dental disease or dental procedures, or by underlying immunosuppressive conditions, and neither had a documented PAVF. Unlike previously reported cases, our patient had no history of dental disease or dental procedures, but had an asymptomatic PAVF as a predisposing factor for hematogenous spread. However, although the patient had poor oral hygiene, indicating a potential source of oral bacteremia, a comprehensive evaluation by a dental specialist was not performed. In addition, although transthoracic echocardiography revealed no abnormalities, transesophageal echocardiography was not performed because the patient was concerned about the procedural risk; therefore, a patent foramen ovale could not be excluded. Although an odontogenic source is plausible, it remains presumptive. The proposed mechanism of hematogenous spread through the PAVF should be regarded as hypothesis-generating rather than definitively established.

The pathogens responsible for brain abscesses associated with asymptomatic PAVF are predominantly oral commensals, with non-oral organisms such as Listeria monocytogenes and Aerococcus viridans being uncommon (14, 15). A 2017 review of the literature on asymptomatic PAVF-related brain abscesses identified 13 cases, with streptococci identified as the most frequently isolated organisms; in six of these cases, cultures yielded no growth (16). Currently reported oral commensals include the Streptococcus anginosus group, the Streptococcus mitis group, Peptostreptococcus species, Bacteroides species, Haemophilus species, and Fusobacterium species (16–19). In the 2022 case of Mycoplasma faucium brain abscess associated with PAVF, C. *rectus* was identified by Next-Generation Sequencing as a component of the polymicrobial flora rather than the predominant pathogen (20); no monomicrobial C. rectus brain abscess has been reported in a patient with either symptomatic or asymptomatic PAVF. To our knowledge, this is the first report of a brain abscess caused solely by *C. rectus* in a patient with PAVF. Our present case broadens the known microbiological spectrum of asymptomatic PAVF-associated intracranial infections and introduces a monomicrobial pathogen-defect pairing that has not been described previously.

One particularly notable aspect of our case is that the pathogen was identified by routine pus culture. A recent retrospective cohort study from a tertiary referral center found that conventional culture detected pathogens in only 50.0% of pus samples from brain abscess (21, 22). For our case, optimized anaerobic culture protocols successfully recovered *C. rectus* from abscess pus. The optimized anaerobic culture protocol consisted of intraoperative pus collection and extended incubation time. Pus was collected intraoperatively and cultured under strict anaerobic conditions with prolonged incubation (up to 4 days), whereas routine anaerobic cultures are commonly finalized after 48 h; transport delays may further reduce the viability of fastidious organisms. This demonstrates that conventional methods remain informative when appropriately applied, even in an era of increasing reliance on molecular diagnostics such as metagenomic next-generation sequencing (21). Previous studies have demonstrated that the recovery rate of anaerobic bacteria is influenced by the culture medium, incubation time, and transport conditions (23, 24). The recovery of *C. rectus* requires appropriate media, a microaerophilic or anaerobic atmosphere, and often, an extended period of incubation (25). Since clinical microbiology laboratories do not routinely implement these conditions, it is highly probable that *C. rectus* is under-diagnosed. When cultures remain negative despite strong clinical suspicion, molecular methods may be considered as a complementary diagnostic approach (5, 21). However, this recommendation is based on the existing literature; in this case, culture successfully isolated C. rectus, whereas molecular methods were not performed because they were not available at the time. Therefore, no direct comparison between conventional culture and molecular diagnostics could be made.

The management of our patient presented a significant clinical challenge. The brain abscess exerted a significant mass effect accompanied by considerable perilesional edema. This created a situation in which the most immediate threat to life was intracranial hypertension rather than the PAVF itself. Surgical intervention is clearly indicated for abscesses causing significant mass effects (26), as the mortality rate can reach up to 64% in patients with signs of herniation (27). Relevant studies indicate that the decision to perform brain abscess excision is mainly based on the location, size, and morphology of the abscess, as well as the presence of severe intracranial hypertension (28). Based on the superficial location of the abscess in the right occipital region and the significant mass effect, surgical resection was selected, which also achieved source control and provided specimens for pathogen identification to guide targeted antimicrobial therapy. Current evidence supports drainage within 24 h of diagnosis when feasible (28); this interval reflected logistical constraints, including the need for neurosurgical consultation, preoperative assessment, and operating room scheduling, rather than a deliberate delay in management.

There is currently no standardized guideline for the antimicrobial treatment of *C. rectus* brain abscess, and the available evidence is mainly derived from case reports and *in vitro* susceptibility studies. Previous studies have shown that *C. rectus* exhibits high *in vitro* susceptibility to therapeutic concentrations of tetracycline, metronidazole, penicillin G, and ciprofloxacin (29). In a 2021 case of *C. rectus* tenosynovitis following a dog bite, broth microdilution susceptibility testing showed low MICs for both meropenem and moxifloxacin (30). Previous case reports of *C. rectus* brain abscess have shown that the isolate was susceptible to amoxicillin-clavulanate, erythromycin, tetracycline, and ciprofloxacin by disk diffusion, and the minimum inhibitory concentration (MIC) of meropenem was determined using gradient strips (0.012 mg/L) (5). After confirming the diagnosis of *C. rectus* brain abscess, in the absence of definitive susceptibility data, we continued intravenous meropenem for 18 days based on previous *in vitro* susceptibility studies, case experience, and its favorable central nervous system penetration. For discharge preparation, after the patient's condition stabilized, we switched to oral moxifloxacin sequentially, considering *in vitro* susceptibility data, case experience, favorable CNS penetration, convenience, and a lower adverse effect profile. According to the ESCMID 2024 guidelines, the recommended antibiotic duration for brain abscess is 6–8 weeks (conditional recommendation, low-quality evidence), and routine oral sequential therapy is not recommended (28). However, in real-world practice, treatment duration is highly individualized, with a median intravenous treatment duration of 33 days, and oral switch has been associated with better neurological outcomes (31, 32). Based on expert opinion, a shorter duration of four weeks may be considered in patients treated with excision of brain abscess (28). The oral sequential therapy used in this case is not routinely recommended by current guidelines, and its efficacy and safety require further validation. In this case, follow-up cranial MRI at week 7 showed radiographic improvement and the patient was asymptomatic; based on the above literature, we assessed the timing of treatment discontinuation.

A 2018 review noted that if PAVF is left untreated, the right-to-left shunt can lead to hypoxemia, paradoxical embolism, stroke, and intracranial abscess (33). Once the intracranial infection is under control, the pulmonary arteriovenous malformation may be considered for interventional embolization to close the shunt and reduce the risk of recurrence (34). However, in this case, the patient declined transcatheter embolization for financial reasons. Continued follow-up is needed to monitor for recurrent brain abscess or paradoxical embolic complications.

This case report has several limitations. First, as a single observational case, it cannot establish a definitive causal relationship between the PAVF and the brain abscess; the proposed hematogenous route remains hypothesis-generating. Second, a formal dental evaluation was not performed, so the odontogenic source remains presumptive. Third, transesophageal echocardiography was not performed, and a patent foramen ovale could not be excluded. Fourth, molecular diagnostics were not available, precluding a direct comparison with culture. Finally, because the patient declined transcatheter embolization, the effect of shunt closure on recurrence risk and prognosis could not be assessed.

## 4 Conclusion

To the best of our knowledge, this case represents the first documented instance of a brain abscess caused by monomicrobial *C. rectus* in a patient with an asymptomatic PAVF. Our experience of this case suggests that the presence of oral commensals in brain abscess cultures may serve as a clue to an underlying anatomical shunt. Even in the absence of respiratory symptoms, PAVF may be considered in cryptogenic cases with brain abscess caused by oral flora.

## Data Availability

The original contributions presented in the study are included in the article/Supplementary Material, further inquiries can be directed to the corresponding author.
